# The List of Available Names (LAN): A new generation for stable taxonomic names in zoology?

**DOI:** 10.3897/zookeys.550.10043

**Published:** 2016-01-07

**Authors:** Miguel A. Alonso-Zarazaga, Daphne Gail Fautin, Ellinor Michel

**Affiliations:** 1 Departamento de Biodiversidad y Biología Evolutiva, Museo Nacional de Ciencias, Naturales (MNCN-CSIC), Madrid, Spain; 2 Department of Ecology and Evolutionary Biology, and Natural History Museum (Biodiversity Institute), University of Kansas, Lawrence, Kansas USA & International Commission on Zoological Nomenclature; 3 Department of Life Sciences, The Natural History Museum, Cromwell Road London SW7 5BD, UK

**Keywords:** Sherborn

## Abstract

The *List of Available Names in Zoology*
(LAN) is an inventory of names with specific scope in time and content, presented and approved in parts, and constituted as a cumulative index of names available for use in zoological nomenclature. It was defined in Article 79 in the fourth edition of the *International Code of Zoological Nomenclature*. The LAN is likely to gain importance with the development of the online Official Registry for Zoological Nomenclature (ZooBank) as it is potentially a source of many nomenclaturally certified names. Article 79 describes the deliberative process for adding large numbers of names to the LAN simultaneously, detailing steps and chronology for submission of a candidate Part to the LAN and consideration of a candidate Part by the public and Commission, but it is largely mute about the contents of a candidate Part. It does make clear that a name within the scope of a Part but not on the LAN has no nomenclatural standing, even if it had previously been considered available, thereby preventing long-forgotten names from displacing accepted ones and the accumulation of *nomina dubia*. Thus, for taxa on the LAN, nomenclatural archaeology – the resurrecting of old unused names to replace by priority names in current usage – will not be worthwhile. Beyond that, it has been unclear if Article 79 is intended to document every available name known within the scope of the Part, or if its intention is to pare the inventory of available names within the scope of the Part. Consideration by the Commission and two committees to deal with the LAN have defined steps to implement Article 79 with the latter intent. Procedures for consideration of a candidate Part are defined in a manual, published as an appendix in this volume.

*List of Available Names in Zoology*

## Introduction

The fourth edition of the *International Code of Zoological Nomenclature* (International Commission on Zoological Nomenclature 1999; hereafter “the Code”) introduced the concept of a *List of Available Names in Zoology*
(LAN) as a way to deal with the plethora of available names that has accumulated over the more than two and a half centuries of zoological nomenclature since the founding datum of the field by Linnaeus’ 10^th^ edition of *Systema Naturae* (1758). The Code defined the LAN as an inventory of names with specific scope in time and content, presented and approved in parts, to constitute a cumulative index of names available for use in zoological nomenclature. The LAN, which was envisioned as a major step in stabilizing nomenclature (cf. Scoble 2004), has taken on additional significance with the development of the online Official Registry for Zoological Nomenclature, with its online presence called ZooBank (http://zoobank.org), because the LAN can potentially serve as a source of many nomenclaturally certified names. It is an idea of long standing, discussed and advanced in fora and articles (e.g., ICZN 1990, Savage 1990), and was welcomed as “the second major change in the Code” in a review of the 4^th^ Edition (Ferraris and Eschmeyer 2000: 908). Polaszek and Michel (2010), for example, proposed that the *Official Lists* and the LAN would play a key role in populating ZooBank.

Article 79 of the fourth edition of the Code describes a procedure for simultaneous addition of large numbers of names to the LAN (the prescription for creating a LAN was novel in the fourth edition). Article 79 deals in considerable detail with who may submit a candidate Part of the LAN for consideration, and with how the candidate Part is to be considered (including timing), but it does not define the content of a candidate Part of LAN. Creating a procedure to implement Article 79 has been the job of a Standing Committee of the *International Commission on Zoological Nomenclature* (hereafter “the Commission”) headed by Commissioner Alonso-Zarazaga, and of an *ad hoc* Committee headed by Commissioner Fautin that was appointed to deal with an application made under Article 79 (as required by Article 79.2.1).

A procedure for adding a Part to the LAN has taken time to establish. A major motive for crafting this procedure clearly, precisely and comprehensively is that it sets precedent. This contrasts with many actions of the Commission. Article 80.5 of the Code, for example, states “An Opinion applies only to the particular case before the Commission and is to be rigidly construed; no conclusions other than those expressly specified are to be drawn from it.” Thus, an Opinion rendered by the Commission applies only to the Case in question, but implementation of Article 79 is a Commission action that stipulates a procedure, and therefore Parts of the LAN adopted by that procedure set precedent.

## Aspects addressed in the Code

Facets of the procedure stipulated in Article 79 for simultaneously adding large numbers of names to the LAN include 1) who may submit a candidate Part for consideration, 2) the scope of a candidate Part, 3) what those who have proposed a Part are to do, and 4) what is to be done with the candidate Part by the Commission.

Submission of a candidate Part of the LAN must be by “an international body of zoologists.” The scope of the candidate Part must be specified in terms of taxon, rank(s), and time period covered. As for Sherborn’s list in *Index Animalium*, the bibliographic source of each name must be provided, but so must details of any relevant actions by the Commission, and the “status” and details of its type, which for species involves citing how type specimens were designated and their repositories. The language of Article 79, therefore, would preclude a latter-day Sherborn: he acted single-handedly, dealt with all animal taxa, and was not concerned with typification.

Article 79 contains considerable detail on timing. Once a candidate Part is open for “comments by zoologists” for 12 months, the community is notified by means of a notice published in the *Bulletin of Zoological Nomenclature*. At the end of the year of review, the *ad hoc* Committee dealing with the particular candidate Part, considering the public input, recommends a vote to the entire Commission. This vote, which must take place no less than two years from the date of publication of the notice (that is, in most cases at least a year after the period of public comment has closed), must be either to abandon further consideration of the candidate Part of the LAN or to consider a candidate Part of the LAN revised in light of comments received; there is no option for the Commission to accept the candidate Part of the LAN at that time. If consideration of the candidate Part continues, another 12-month period of public input on the revised candidate Part follows (subsequent to notice), after which the *ad hoc* Committee again recommends a vote to the entire Commission. This vote, too, must take place no less than two years from the date of publication of the notice, but this vote is either to abandon further consideration of the candidate Part or to accept it. Thus, the entire process of considering a candidate Part that is eventually approved for inclusion in the LAN takes a minimum of four years. When the Commission votes to add a candidate Part to the LAN, notice to that effect must promptly be published in the *Bulletin of Zoological Nomenclature*.

The Code also comments on what the LAN is not. Article 80.8 distinguishes between the LAN and the *Official Lists*, of which there are four, as defined in the Glossary, the relevant ones being the *Official List of Family-Group Names in Zoology*, the *Official List of Generic Names in Zoology*, and the *Official List of Specific Names in Zoology*. These lists are compiled by the addition of names singly or at most in small numbers, by contrast with the LAN, which is assembled by simultaneously adding large numbers of names. In case of conflict between the status of a name as given in the LAN and on one of the *Lists*, the former takes priority, as it does also in case of conflict between the status of a name as given in the LAN and an Opinion of the Commission (Article 80.8). However, although the LAN supersedes other actions of the Commission, according to Article 79.5, “If there are exceptional circumstances and only when an entry in the *List of Available Names in Zoology* is a cause of confusion, the Commission may amend the entry by use of its plenary power and publish its ruling in an Opinion.” Nonetheless, some confusion still exists about these fundamentally different documents, the LAN and the *Official Lists* and *Indexes* (e.g. Gregory 2010).

## Aspects not addressed in the Code

Despite details of who may propose a candidate Part and stipulating actions along a time line leading to rejection or adoption of a candidate Part, the Code provides few details about the desired contents of a candidate Part of the LAN. Article 79.4.3 does state “No unlisted name within the scope (taxonomic field, ranks, and time period covered) of an adopted Part of the *List of Available Names in Zoology* has any status in zoological nomenclature despite any previous availability.” Thus, any name discovered subsequent to the adoption of a Part of the LAN does not compete for priority, etc., so Article 79 has the effect of preventing nomenclatural archeology as an end rather than a means on one side and getting rid of *nomina dubia* on the other. Of course, any omission or error can become the subject of an appeal to the Commission under Article 79.5 or 79.6.

**Figure 1.**
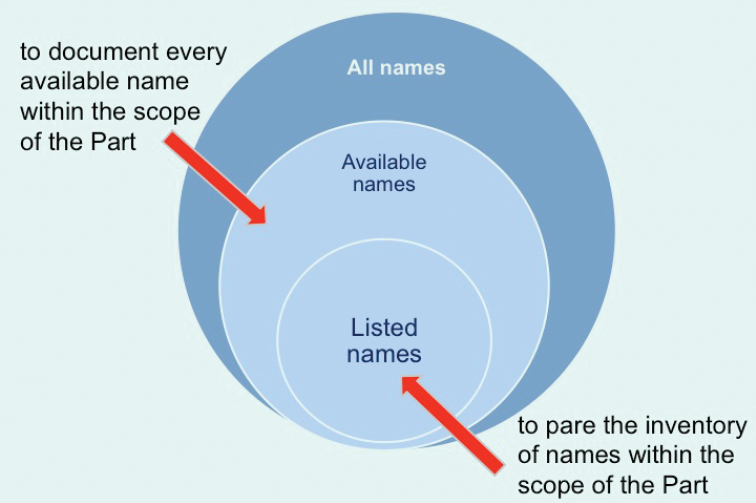
A schematic representation of the universe of animal names, the two possible choices in compiling a LAN and the contents of the two resulting lists.

Central to defining the content of a candidate Part of the LAN is understanding of what the LAN is meant to be. The main choices are whether the LAN is intended to contain all available names known or only some (carefully selected) available names; both positions have been advocated to us by current and past Commissioners. The universe of names of animals includes vernacular names, manuscript names, names decreed by the Commission to be unavailable for a diversity of reasons, etc. These are represented by the largest circle in Figure 1. Available names are a subset of all names, represented by the intermediate circle in Figure 1. If the LAN is to be an inventory of all available names, assembling it is a purely bibliographic exercise that seeks to uncover every name ever made available. The innermost circle in Figure 1 represents the subset of available names that are potentially valid. Nor does this inventory include names of doubtful application – those termed *nomina dubia* in the glossary of the Code. By this reasoning, junior objective synonyms and primary homonyms are to be excluded from the LAN, but it must include names considered to be subjective synonyms (they are still available and are retained in case they are needed in the future, e.g., for cryptic species).

What names belong on the LAN? What did the framers of Article 79 have in mind by establishing the elaborate procedure to assemble the LAN described in the Code? The first principle stated in the Introduction to the fourth edition of the Code is “The Code refrains from infringing upon taxonomic judgment, which must not be made subject to regulation or restraint,” so assembling the LAN cannot involve excluding any available names based on taxonomy.

Retaining all available names – that is, holding on to names the significance of which may never be known – risks a Type I error: we must continue to inventory and deal with names of uncertain application in the hope we might someday associate at least some with a taxon. Paring names to those that we can currently associate with taxa risks a Type II error: we might discard a name that could eventually be associated with a taxon. Which type of error is the more costly involves considering the effort involved both in rectifying and in not rectifying the error. Retaining all available names requires not only continuing to inventory many names of uncertain application, but also, if the taxon to which a hitherto doubtful name applies is determined, redefining and retypifying the name, effectively redescribing the taxon. Restricting the LAN to a subset of all available names means effort need not be expended in carrying along names of uncertain application, but if a taxon that had had a name is rediscovered, that taxon must be described, with a new name assigned to it. This seems to us the more parsimonious procedure.

We have come down on the side of the LAN being a subset of the known available names. We consider that the taxon with a name that would not merit placement on the LAN would have to be redescribed extensively to fix the name, associating it unambiguously with the concept (and thereby meriting placement on the LAN); this would require as much effort as describing the taxon anew. Moreover, discarding the old name would not be disruptive because that name would not have been unambiguously used for the taxon, at least in a very long time. Thus paring the list will ultimately save effort, including the increasingly precious time of taxonomists. This interpretation is consistent with Article 79.4.3 (“No unlisted name within the scope (taxonomic field, ranks, and time period covered) of an adopted Part of the *List of Available Names in Zoology* has any status in zoological nomenclature despite any previous availability”), with Recommendation 79A (“If for taxonomic and historical purposes an author desires to cite a name that is no longer available because it is not included in the relevant Part of the *List of Available Names in Zoology* adopted by the Commission, it should be made clear that it no longer has a status in zoological nomenclature”), and with item 15 of the Introduction (“Names within the scope of such an adopted list but not listed in it will be treated as unavailable”). Some names that were previously available will not end up on the LAN. In this way, each Part of the LAN will become a new datum for zoological nomenclature. This is similar to what the microbiologists did in making a new start for bacterial nomenclature on 1 January 1980, although, of course, Parts of the LAN will take effect at different times (Sneath 2003). Once accepted according to Article 79, the names on the Parts of the LAN can be entered into ZooBank with the assurance that they have been certified through a lengthy process of public vetting. If mandatory registration becomes part of making a name or act available (e.g., Krell 2009), ZooBank would achieve the same economy of effort that the Bacteriological Code has effected.

Article 79 lays out the requirements and timing of consideration of a candidate Part of the LAN. Implementation of Article 79, including stipulation of the contents of the candidate Part, are the subject of a Manual developed by the Standing Committee headed by Commissioner Alonso-Zarazaga, approved in the ICZN Session of November 20^th^ 2013 held in Singapore, that accompanies this article as an Appendix and that will be posted on the Commission website (http://iczn.org).

The lengthy vetting process helps minimize the risk of a name in wide use inadvertently being omitted from the LAN. However, should the process fail, Article 79.6 provides that “If the Commission determines that there is a previously available name within the scope of an adopted Part of the *List of Available Names in Zoology* that has been omitted from the *List*, in exceptional circumstances the Commission may by use of the plenary power add an appropriate entry to that Part of the *List* and record this in an Opinion. The availability of the name thereby becomes restored.”

An inventory that constitutes the candidate Part of the LAN may contain all available names within the scope of the Part. A name not on the candidate Part may have been intentionally excluded, or it may simply have been overlooked. So that members of the public reviewing such an inventory can distinguish between these two possibilities, the implementation document for Article 79 includes the requirement that the compilers of a candidate Part of the LAN include also an inventory of available names they do not want placed on the LAN. The form of this inventory is not stipulated, but the names in the two categories must clearly be differentiated: there may be two separate lists or the names may be on a single list but be distinguished by typeface, an indication such as an asterisk, etc. This inventory of names will become part of the public nomenclatural record – but these names, under Article 79.4.3 have no “status in zoological nomenclature despite any previous availability.” Some concern has been raised about the minimum requirements that names placed in the list to be deleted should meet (e.g. Eschmeyer 2003, Dayrat 2005 and references therein) and also about how the placement of senior homonyms on it will affect junior homonyms in other taxonomic groups, for which there is no rule at present.

The LAN was designed to help stabilize nomenclature and to relieve the burden on taxonomists of dealing with names of uncertain application that have accumulated during more than two centuries of modern taxonomic science. A side benefit is that the LAN would deter the practice of what has been termed “taxonomic vandalism” (Polaszek 2010). It allows dealing with large numbers of homonymy and priority problems at the stroke of the pen: one by one, they would take a very long time and much more effort both for the proponents and for the Commission (cf. Bouchard et al. 2011). Stability achieved this way would be effective and everlasting. Creation of this kind of list is time-consuming, requiring that all the relevant literature be checked (Steiner and Kabat 2004), and so is not currently academically rewarding. Taxonomy would benefit greatly if institutional recognition were given for the considerable and lasting value of work on a LAN Part. No Part of the LAN has yet been approved as of writing this paper: the Part on the Species Group Names of Phylum Rotifera (Segers et al. 2012) is still being considered; two others on Family and Genus Group Names in Aphidoidea are at an impasse for submission.

Thus, while the LAN is ultimately more than a modernised version of Sherborn’s *Index Animalium*, it also meets the need for creating order among an array of names with very different levels of usage and taxonomic effectiveness (Evenhuis 2016). This is a necessary step in streamlining taxonomic work (Lyal 2016, Page 2016, Penev et al. 2016) and allowing organismal names to efficiently function as handles for all biological information (Pyle 2016).
